# Short-term outcome of levamisole in frequently relapsing nephrotic syndrome: a single-center prospective cohort study

**DOI:** 10.3389/fneph.2025.1539776

**Published:** 2025-03-21

**Authors:** Sabeeta Khatri, Irshad Ali Bajeer, Aasia Zubair, Ali Asghar Anwar Lanewala, Seema Hashmi

**Affiliations:** Department of Pediatric Nephrology, Sindh Institute of Urology and Transplantation, Karachi, Pakistan

**Keywords:** frequently relapsing nephrotic syndrome, levamisole, childhood, outcome, prospective

## Abstract

**Introduction:**

This study aims to describe the outcome of levamisole (LEVA) treatment in children with frequently relapsing nephrotic syndrome (FRNS).

**Methods:**

This prospective cohort study was conducted at the Department of Pediatric Nephrology, Sindh Institute of Urology and Transplantation from 1 January 2019 to 31 December 2020. Children aged 1–18 years diagnosed with FRNS were included. LEVA was started with a dose of 2–2.5 mg/kg every other day for 2 years along with low-dose prednisolone in the first year.

**Results:**

A total of 70 children with FRNS were enrolled in the study. The median age was 7.5 [interquartile range (IQR) 5.0–9.6 years] with a slight predominance of boys (42, 60%). The mean number of relapses and cumulative dose of steroids significantly decreased after 2 years of LEVA therapy and during the 1-year follow-up. LEVA non-response was observed in half of the studied participants (28, 46%). The responders and non-responders were comparable in terms of cumulative dose of steroids and number of relapses in the year prior to starting LEVA [5,242 ± 1,738 versus 4,910 ± 1,469 (p-value = 0.52) and 5.4 ± 2.4 versus 5.2 ± 2.1 (p-value = 0.85)].

**Conclusion:**

LEVA therapy resulted in a substantial reduction in the frequency of relapses and cumulative dosage, indicating its potential as an alternative option for children with relapsing disease.

## Introduction

Idiopathic nephrotic syndrome is the most common glomerular disease in children, affecting 2.92/100,000 children per year (1). Fortunately, 80%–85% of children respond to corticosteroid therapy at the first presentation (2). However, nearly 50% keep relapsing with either a steroid-dependent or frequently relapsing pattern (3). This cohort of children is at risk of steroid toxicity and needs sparing therapies to avoid deleterious effects (4).

The choice of a second-line agent has been long debated. In 2020, the Cochrane systematic review concluded that the choice between levamisole (LEVA), mycophenolate mofetil, calcineurin inhibitors, and cyclophosphamide must be left to physician preference (5). The Kidney disease improving global outcomes (KDIGO) guidelines in 2021 suggested the use of LEVA and cyclophosphamide specifically in frequently relapsing nephrotic syndrome (FRNS) (6). The Indian Society of Pediatric Nephrology (ISPN) recommends LEVA when there is a relapse on steroids of less than 1mg/kg on alternate days, no complicated relapse, and development of steroid toxicity (7). The duration and frequency (daily versus alternate day) of therapy with LEVA are also debated (8).

LEVA is an anthelminthic drug that has been used in the treatment of nephrotic syndrome since the 1980s (9). Apart from the modulation of the immune system with its alteration of T and B cells, LEVA has shown a direct effect on podocytes to achieve remission (10). LEVA offers the added advantage of fewer adverse effects and lower cost (11). Kuzma-Mroczkowska et al. showed that LEVA leads to a reduction in the number of relapses in both steroid-dependent and frequently relapsing nephrotic syndromes (12). Kiruba et al. has shown better efficacy of LEVA in children with frequently relapsing nephrotic syndrome (13).

In general, our children receive a high dose of steroids because of multiple relapses, and using LEVA could also postpone the introduction of CNI, thereby reducing the risk of its potential nephrotoxicity. We present the results of a prospective cohort study documenting the outcomes of a 2-year alternate-day LEVA therapy in children with frequently relapsing nephrotic syndrome.

## Materials and methods

This prospective cohort study utilized non-probability convenience sampling and was conducted at the outpatient Department of Pediatric Nephrology, Sindh Institute of Urology and Transplantation, Karachi. The study received approval from the ethical review committee. All children diagnosed with frequently relapsing nephrotic syndrome between 1 January 2019 and 31 December 2020 were enrolled.

Children aged 1 to 18 years with FRNS who had no prior use of immunosuppression drugs and were in complete remission at the time LEVA was prescribed were included in the study. The exclusion criteria encompassed children with steroid-dependent nephrotic syndrome, primary or secondary resistant nephrotic syndrome, or infantile or congenital nephrotic syndrome.

Before enrolling participants in the study, the diagnosis of FRNS, treatment protocol, and monitoring during therapy were established and published as per the national consensus recommendations (14). FRNS was defined as two relapses within 6 months of initial response with steroids or four relapses in any 12-month period (12). The relapse was treated with prednisolone 2 mg/kg/day for 2 weeks and then 1.5 mg/kg/day for 4 weeks. Complete remission was achieved before initiating LEVA.

LEVA was started with a dose of 2-2.5mg/kg on alternate days for 2 years. Steroids were tapered by 0.5 mg/kg every 2 weeks and a minimal dose of 0.25 mg/kg was continued for the first year. The children were monitored with complete blood count and liver function tests. Relapses during the study period were treated with the standard duration of steroid therapy, after which the original study protocol was resumed. Children who experienced two relapses within any 6-month period of the study were classified as LEVA non-responders, and an alternative immunosuppressant drug was considered.

A structured proforma was used to document the baseline demographics, duration since diagnosis of nephrotic syndrome, and pattern of FRNS (two relapses in 6 months or four in 12 months). Data on cumulative steroid exposure were collected for the year preceding the FRNS diagnosis, during the 2-year study period, and for 1 year after completing therapy.

The total number of children with FRNS seen in our institute in a year is 70. Based on an estimate of the efficacy of the alternate-day LEVA therapy of 82% with a margin of error of 5% and a 95% confidence level, a total of 55 patients were required. However, assuming that 5% would be lost to follow-up, a total of 60 patients was needed for this study (13).

SPSS version 26 was used for the statistical analysis. Normally distributed continuous variables were expressed as means with standard deviations and the rest as medians with interquartile ranges (IQRs). The qualitative variables were reported as percentages or ranges. Appropriate tests of significance were applied. A p-value < 0.05 was considered significant.

## Results

**Figure 1:**
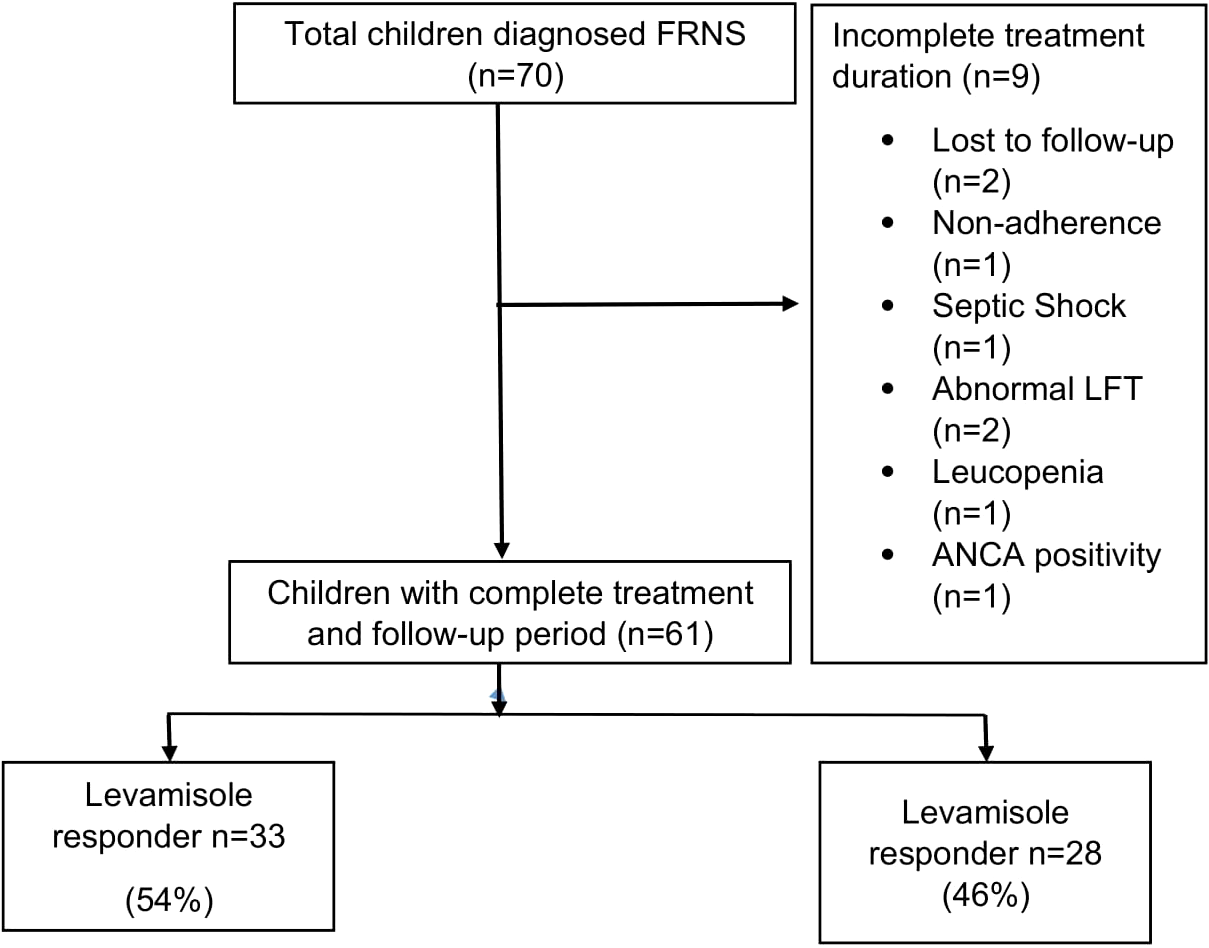
Flowchart depicting the inclusion of children in the final analysis.

We prospectively enrolled 70 children with FRNS in the study. Of these, 9 (13%) could not complete the treatment protocol due to the reasons mentioned in **Figure 1**.

The baseline characteristics of the study cohort were comparable and are described in **Table 1**.

**Table 1:** Comparison of levamisole responders and non-responders.

Parameter	LEVA - non-responder (n=28)	LEVA responder (n=33)	P-value
Median Age in years (Interquartile range)	7 (5–8)	9 (6–10)	0.06
Sex (Boys)	18 (64%)	18 (54%)	0.30
Median duration of disease in months (Interquartile range)	17.5 (12.2–35.5)	22 (15–50)	0.87
Pattern of relapse (2 relapses in 6 months)	22 (78%)	30 (91%)	0.16
Number of relapses before LEVA per year	5.4 ± 2.4	5.2 ± 2.1	0.85
Dose of LEVA During therapy (mg/kg)	2.3 (2–2.5)	2.2 (2–2.5)	0.75
Cumulative dose of steroids one year prior to LEVA (mg/m^2^)	5242 ± 1738	4910 ± 1469	0.52

LEVA, levamisole; mg, milligram.

The median age was 7.5 (IQR: 5–9.6 years) with a slight predominance of boys (42, 60%). The majority of the children (57, 81.4%) were diagnosed with FRNS with the pattern of four relapses in a 12-month period. The median duration of nephrotic syndrome prior to enrollment in the study was 20 (IQR: 12.7–40.2 months) and the prescribed dose of LEVA was 2.3 (IQR: 2–2.5 mg/Kg) alternate day.

**Figure 2:**
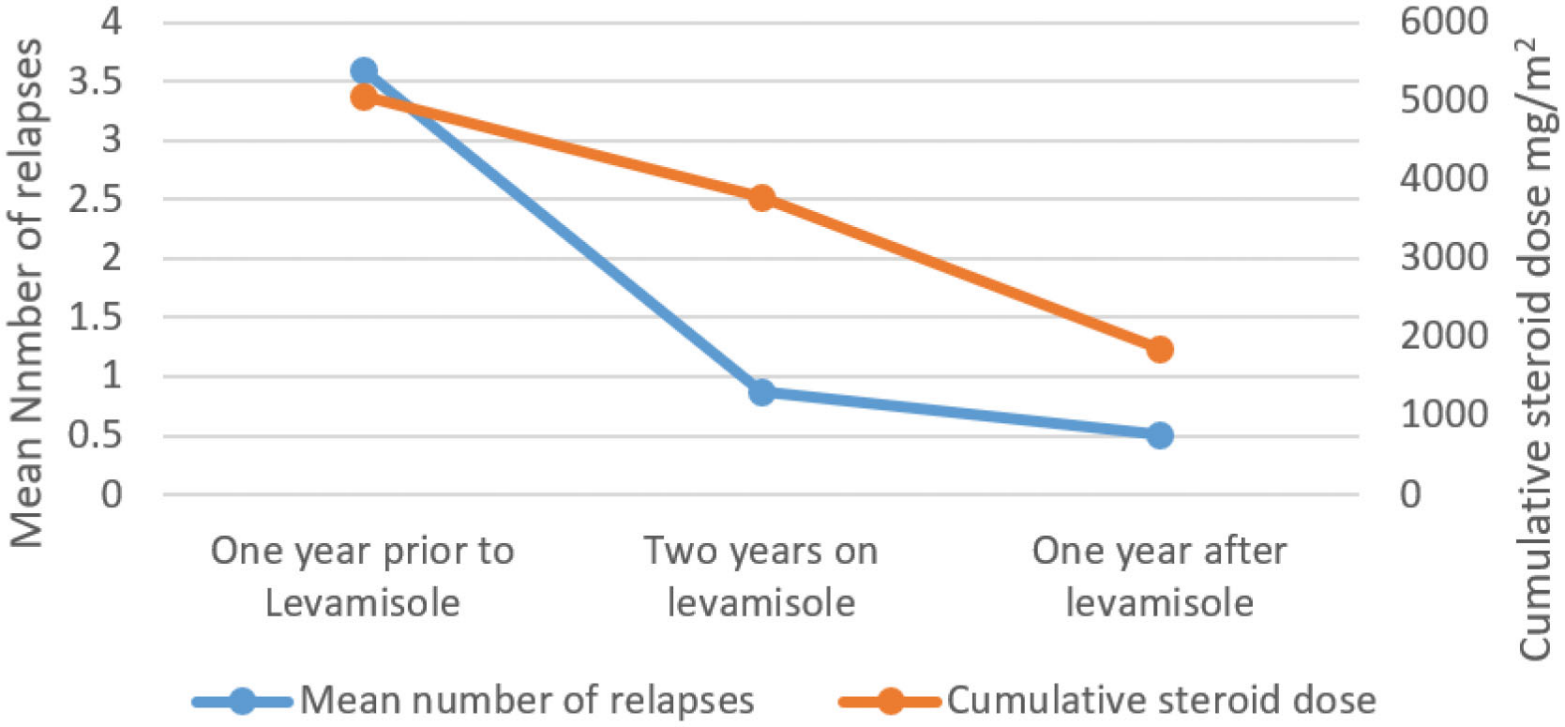
Effect of Levamisole on cumulative dose of steroids and mean number of relapses in the study cohort.

In the study cohort, 61 children completed 24 months of LEVA therapy and a 12-month follow-up period. The number of relapses significantly decreased in the period from 1 year before starting LEVA to during treatment either with LEVA and steroids or LEVA without steroids, with means of 3.6 ± 0.78, 0.87 ± 0.86, and 0.52 ± 0.73, respectively (p-value = 0.00). Similarly, the cumulative dose of steroids in the preceding year before starting LEVA was 5,067 ± 1,396 mg/m^2^. This decreased to 3,785 ± 1,636 mg/m^2^ during the 2 years of therapy, and further decreased to 1,856 ± 1,627 mg/m^2^ in the year following completion of LEVA (p-value = 0.00). This has been graphically shown in **Figure 2** and the analysis excluded children with no response to LEVA.

Non-response to therapy showed no significant association with gender (18 boys versus 10 girls, p = 0.30), age under 7 years (16 versus 12, p = 0.13), or a duration of more than 24 months from nephrotic syndrome diagnosis to the development of FRNS (16 versus 12, p = 0.42).

The majority of LEVA non-responders (23, 82%) required oral cyclophosphamide as an additional drug to achieve remission. Four children (14%) developed secondary steroid resistance and required a kidney biopsy which showed minimal change disease; cyclosporine was prescribed for this. Although one child (4%) was categorized as a LEVA non-responder, he attained sustained remission prior to the initiation of further immunosuppression. Despite administration of cyclophosphamide, 12 (52%) continued to relapse, with infrequent relapse patterns in 7 (58%) and persistent FRNS in 5 (42%), who subsequently required renal biopsies and treatment with calcineurin inhibitors.

LEVA showed a relatively better response in 33 (54%) children at the end of the therapy. In the 12-month follow-up period, 17 (52%) showed sustained completed remission, 9 (27%) had an infrequently relapsing course, and 6 (18%) demonstrated FRNS for which oral cyclophosphamide was administered. One child (3%) presented with secondary SRNS, a kidney biopsy showed FSGS, and cyclosporine was started.

Nine children (13%) could not complete the study protocol and follow-up period. Three (34%) were lost to follow-up and one (11%) showed non-adherence, so cyclophosphamide was prescribed considering the shorter duration of therapy. Complicated relapse of NS with septic shock was seen in one patient (11%). Reversible derangement of liver function tests (negative hepatitis A and E profiles) and leucopenia were documented in two (22%) and one (11%) children respectively. A 4-year-old boy (11%) presented at 18 months of therapy with malaise, generalized bodyache, and non-specific joint pain for a few days. He had normal renal function, his urine was negative for protein and RBCs, and ANA and rheumatoid factor were negative. A further workup revealed ANCA MPO positivity. LEVA was discontinued and analgesics were advised. During subsequent follow-ups, his symptoms resolved within 4 weeks and ANCA MPO titers gradually declined to negative in 12 months; he remained in sustained remission at 36 months.

## Discussion

The steroid-sparing effect of LEVA has been utilized for a long time. Our study selected a homogenous population of children with FRNS and prescribed a uniform protocol for 2 years along with 1-year follow-up after completion of the therapy. The median age of the study participants was 7.5 years (5 – 9.6) with a slight predominance of boys (42, 60%). The age is comparable to the regional data of Sinha et al. However, they had a higher proportion of boys (85%) (14). The median duration of nephrotic syndrome prior to starting LEVA was 20 months (12.7–40.2 months), which is a notably longer period than the multicenter randomized trial conducted by Gruppen et al., where they observed for 12 months (15). This correlates with the majority of our cohort being categorized as FRNS on the basis of four relapses in 12 months (57, 81%).

We prescribed LEVA for a total duration of 24 months and a similar duration was studied by Kiruba et al. and Ekambaram et al. (13, 16). Nonetheless, the duration of LEVA is variable in various other studies (15, 17). Even the recent guidelines on steroid-sensitive nephrotic syndrome lack uniformity on the duration of LEVA (6, 7, 18, 19). However, the ISPN advocates LEVA a dose of 2–2.5 mg/kg every other day (EOD) for 2–3 years (7). The most commonly studied dose of LEVA is 2–2.5 mg/kg EOD and we prescribed a median dose of 2.2 mg/Kg EOD (IQR: 2–2.5). Recently, there has been an inclination towards a daily dose of LEVA, especially in steroid-dependent FRNS and non-responders to the EOD schedule (17, 20). However, none of the international guidelines have recognized it so far. The concomitant administration of steroids with LEVA is very inconsistent and we continued low-dose steroids in the first year only. The cumulative dose of steroids prescribed in our study is comparable to other studies, as shown in **Table 2**.

**Table 2:** Comparison of cumulative dose of steroids.

Author/Year	Pre-LEVA	On LEVA	Post-LEVA
Sabeeta et al., 2025, mg/m^2^	5,067 ± 1,396	3,785 ± 1,636	1,856 ± 1,627
Khemchand/2020, mg/pt mean (18)	3,389.81 ± 2,785.22	2,471.97 ± 2,024.98	661.37± 905.37
Kiruba/2017, mg/m^2^ median (13)	4,200 (3,200, 4,300)	1100 (500, 2,900)	2,800 (1,375, 4,200)
Ekambaram/2014, mg/m^2^ mean (17)	4,109.29 (1154)	2,491.8 (694)	660.7 (10.7)
Elmas/2013, mg/m^2^/year (24)	5,582.0 (2,137.0–17,340.0)	2,166.0 (840.0–9,325.0) mg/m2	–
Boyer/2008, (n=10), mg/m^2^/year (25)	5,717	2,612	881
Alsaran/2006, mg/m^2^/month (22)	548 ± 198	255 ± 157	–

We observed a significant reduction in exposure to steroids with LEVA treatment. Alsaran et al. demonstrated a proportionately greater reduction from 4,100 mg/m^2^ to 900 mg/m^2^. Similarly, the mean number of relapses prior to the LEVA in our study was 3.6 ± 0.78 and this reduced to 0.52 ± 0.73 (p-value 0.00) in the year after completion of the therapy. Our cohort exhibited a relatively better frequency of relapses compared to Alsaran et al. and Kiruba et al., as they reported 1.6 and 1.1 relapses during therapy, respectively (13, 21). This may be due to prolonging steroid therapy by a year in our protocol.

Of the children who completed the study, LEVA therapy resulted in responses in 33 (54%), and the rest (28, 46%) were categorized as non-responders. Moorani et al. reported a sustained remission rate of 76%, while Grupen et al. documented a 26% rate at 1 year, and Sinha et al. reported a rate of 34% (14, 15, 17). The wide discrepancy in the outcome may be due to the selection of patients (FRNS versus SDNS), concomitant administration of steroids, and duration of LEVA therapy. The response and non-response groups of children in our cohort to LEVA were comparable in terms of age, dose of LEVA, and cumulative doses of steroids.

Out of the nine (13%) who failed to complete the study protocol, three (33%) experienced drug effects. One child developed a transient rise in transaminases, while another experienced a rise in leucopenia. Although these children had reversible effects, as per our study protocol, we decided to switch them to another steroid-sparing agent. Abygunawardena et al. reported three children with deranged liver functions and one with neutropenia in a cohort of 72 children (20). One of our children with MPO-positive ANCA was identified through non-specific symptoms such as arthralgia and malaise. He developed symptoms after 12 months of therapy, highlighting that, although rare, long-term use can lead to ANCA positivity (22, 23). The same pattern was highlighted in the trial conducted by Grupen et al. (15).

The IPNA consensus guidelines now recommend 6-monthly monitoring of ANCA profile when on LEVA therapy (19).

Our study has certain limitations, such as its single-center, non-randomized design and a 13% attrition rate, which raise questions about its internal validity. Additionally, no distinction was made between relapses occurring spontaneously and those triggered by infections. However, the study’s strength lies in selecting a naive cohort of children with FRNS who had never been exposed to other steroid-sparing drugs. They were treated with a uniform protocol and prospectively followed-up.

In conclusion, our study demonstrated a positive response to LEVA with a significant reduction in steroid exposure and fewer relapses. This underscores the potential of LEVA as a steroid-sparing agent for frequently relapsing nephrotic syndrome. Further research with refined patient selection criteria is needed to optimize outcomes and clarify LEVA’s therapeutic role in this patient population.

## Data Availability

The raw data supporting the conclusions of this article will be made available by the authors, without undue reservation.
